# Local and Remote Ischemic Preconditioning Improves Sprint Interval Exercise Performance in Team Sport Athletes

**DOI:** 10.3390/ijerph182010653

**Published:** 2021-10-12

**Authors:** Ching-Feng Cheng, Yu-Hsuan Kuo, Wei-Chieh Hsu, Chu Chen, Chi-Hsueh Pan

**Affiliations:** 1 Department of Athletic Performance, National Taiwan Normal University, Taipei 11677, Taiwan; 2 Sports Performance Lab, National Taiwan Normal University, Taipei 11677, Taiwan; hsu.w.c1982@gmail.com (W.-C.H.); alvinc5939@gmail.com (C.C.); jasonpan8011@gmail.com (C.-H.P.); 3 Department of Physical Education, Chinese Culture University, Taipei 11114, Taiwan; gyx2@ulive.pccu.edu.tw; 4 Graduate Institute of Sports Training, University of Taipei, Taipei 11153, Taiwan; 5 Department of Physical Education and Sport Sciences, National Taiwan Normal University, Taipei 10610, Taiwan

**Keywords:** anaerobic capacity, blood flow occlusion, fatigue resistance, high-intensity interval training

## Abstract

The aim of this study was to investigate the effects of local (LIPC) and remote (RIPC) ischemic preconditioning on sprint interval exercise (SIE) performance. Fifteen male collegiate basketball players underwent a LIPC, RIPC, sham (SHAM), or control (CON) trial before conducting six sets of a 30-s Wingate-based SIE test. The oxygen uptake and heart rate were continuously measured during SIE test. The total work in the LIPC (+2.2%) and RIPC (+2.5%) conditions was significantly higher than that in the CON condition (*p* < 0.05). The mean power output (MPO) at the third and fourth sprint in the LIPC (+4.5%) and RIPC (+4.9%) conditions was significantly higher than that in the CON condition (*p* < 0.05). The percentage decrement score for MPO in the LIPC and RIPC condition was significantly lower than that in the CON condition (*p* < 0.05). No significant interaction effects were found in pH and blood lactate concentrations. There were no significant differences in the accumulated exercise time at ≥80%, 90%, and 100% of maximal oxygen uptake during SIE. Overall, both LIPC and RIPC could improve metabolic efficiency and performance during SIE in athletes.

## 1. Introduction

In 2010, de Groot et al. [1] extended the concept of ischemic preconditioning (IPC), which involves brief cycles of ischemia and reperfusion, from the medical field to the sports science field, and discovered that IPC could help increase maximal oxygen uptake ($\dot{V}$O_2_max). Since then, many studies have explored the benefits and possible mechanisms of IPC on sports performance. IPC can be divided into local (LIPC) and remote (RIPC) ischemic preconditioning according to its location of function. The difference between the two approaches is whether blood flow is occluded in the same muscle group that is subsequently used during exercise. For example, for cycling exercise, LIPC refers to application of blood flow occlusion to the legs, whereas RIPC refers to application of blood flow occlusion to the arms. At present, it is thought that both LIPC and RIPC may improve functioning of the mitochondrial ATP-dependent potassium channel, attenuate ATP depletion, promote phosphocreatine (PCr) resynthesis, enhance metabolic efficiency, and increase vasodilation, oxygen delivery and extraction, thereby enhancing subsequent endurance performance [2,3]. However, the acute effect of IPC on the performance of repeated or interval sprints, in which aerobic contribution is predominant [4], remains controversial.

Some studies have suggested that LIPC can improve subsequent repeated sprint performance, including the 12 × 6-s cycling sprint [5], 3 × (6 × [15 + 15-m]) shuttle sprints [6], and 6 × 50-m swimming sprint [7]. However, others have reported the opposite, i.e., that for repeated sprints with a short overall exercise time, including 5 × 6-s [8] and 10 × 6-s cycling sprints [9], LIPC does not enhance performance. Alternatively, application of LIPC to single short-duration sprints, such as 10–30-m sprints, revealed no obvious benefits [10,11]. Some studies have indicated that LIPC may be beneficial only in single sprints powered by glycolysis, such as 50-m swimming sprint [12] and 60-s cycling sprints [13]. In contrast, a study by Paixao et al. [14] has reported that LIPC cannot improve the power output of three sets of 30-s Wingate-based sprint with 10-min rest intervals. During a single 30-s Wingate-based sprint, PCr availability can be restored to 65% of the rest value after 1.5 min of post-exercise rest, or 86% after 6 min of post-exercise rest [15]. Therefore, LIPC cannot enhance the performance of repeated 30-s Wingate-based sprints, probably because most PCr availability can be restored through passive recovery during long rest intervals. It would be relevant to explore whether LIPC can improve the performance of the subsequent 4–6 sets of 30-s Wingate-based sprint interval exercise (SIE) with short (4-min) rest intervals, as it is an exercise intervention commonly adopted to improve aerobic capacity and performance in untrained and trained individuals [16].

For the RIPC research, some studies have suggested that RIPC does not enhance aerobic or anaerobic performance in exercises such as the 1-h cycling time trial [17], 7 × 200-m incremental swimming test [18], and 6 × 6-s and 30-s cycling sprint [19]. On the other hand, others have reported that RIPC can help to shorten the time needed for swimming 100 m [18] and can extend the time to task failure during handgrip exercise [20]. More recent studies have indicated that RIPC can increase the number of repetitions and total training volume during resistance exercise [21,22]. Kraus et al. [23] pointed out that RIPC of bilateral rather than unilateral upper limbs can increase the power output during four sets of 30-s Wingate-based SIE. They also suggested that inconsistent results may be related to the number of muscle groups involved during the application of IPC. Therefore, whether the acute effect of LIPC on the performance of SIE is superior to that of RIPC requires further clarification.

For team-sport athletes, sprint and high-intensity interval training programs have been shown to be effective approaches to enhance aerobic capacity and performance [24]. Coaches and sports scientists often find ways to further improve training quality and training adaptation. Therefore, the purpose of this study was to investigate the acute effects of LIPC and RIPC on SIE performance in athletes. We hypothesized that both LIPC and RIPC could improve SIE performance, in terms of both power output and fatigue resistance, and that LIPC would be more effective than RIPC in this respect. We also hypothesized that LIPC and RIPC could increase metabolic efficiency during SIE.

## 2. Materials and Methods

### 2.1. Participants

Fifteen male Division I collegiate basketball players (age 21 ± 2 years; height 1.87 ± 0.08 m; body mass 86 ± 13 kg; $\dot{V}$O_2_max, 51.9 ± 7.8 mL·kg^−1^·min^−1^; blood pressure 126/68 mmHg) were recruited to complete this repeated measured crossover study. The priori power analysis (G*Power 3.1.9.4) was used to calculate the appropriate sample size. Based on a previous meta-analysis study [3] on the overall impact of IPC on exercise performance (effect size = 0.43), the minimum sample size of *n* = 9 was required for this study (α = 0.05, β = 0.20). All participants completed a medical history and health questionnaire, and signed informed consent forms before participating in the experiment. The participants refrained from drinking alcoholic or caffeinated beverages for 24 h before the experiments began and fasted for at least 4 h prior to visiting the laboratory, in order to reduce the interference of food in the experiment. The study was conducted according to the guidelines of the Declaration of Helsinki, and approved by the Research Ethics Committee of National Taiwan Normal University, Taipei, Taiwan (Approval code: 201612HM010).

### 2.2. Experimental Design and Protocols

**Figure 1:**
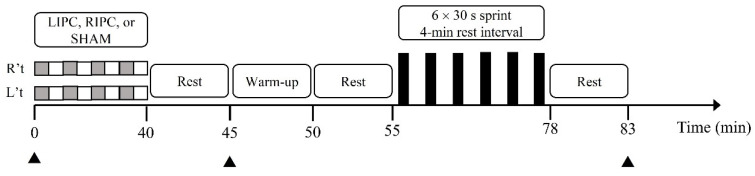
Protocol schematic. R’t, right thigh or upper arm; L’t, left thigh or upper arm; LIPC, local ischemic preconditioning; RIPC, remote ischemic preconditioning; ■, Occlusion; □, Reperfusion; ▲, Blood lactate and pH value.

After a familiarization trial, participants performed an incremental cycling test (GXT) and two control (CON) trials, separated by at least 3 days, on a cycling ergometer (Cyclus 2, RBM Elektronik, Automation, Leipzig, Germany). The cycling ergometer was equipped with an electromagnetically-braked system. During the subsequent visits, participants underwent LIPC, RIPC, or sham (SHAM) treatment in a randomized crossover design, separated by at least 4 days, before conducting six sets of 30-s Wingate-based SIE test (Figure 1). When they arrived at the laboratory, their body mass was measured to determine the load of the SIE test. Before treatment, participants were asked to rest supine for 10 min for baseline measurements, including their heart rate, blood lactate concentration, and pH level. The heart rate and oxygen uptake ($\dot{V}$O_2_) were continuously measured during the SIE test. Blood samples for pH and lactate concentrations were drawn before (baseline) and 5-min after treatment, and 5-min after the SIE test. The participants completed all of the trials during the same time-period (±2 h) of testing days to eliminate any effect of circadian variation.

### 2.3. Incremental Cycling Test

During each participant’s first visit to the laboratory, the cycling ergometer seat and handlebars were adjusted for comfort. These same settings were restored for each consecutive exercise trial. Before the GXT, all participants were asked to cycle at different pedaling rates (70, 80, and 90 rpm) for a while (~1 min), and then chose the most comfortable rate as their self-selected cadence. Participants first performed 3 min of unloaded baseline pedaling (0 W); the load was increased by 30 W every min thereafter until volitional exhaustion. During the GXT, the participants maintained a self-selected cadence for as long as possible. Strong verbal encouragement was provided throughout the trial. Exhaustion was defined as a pedaling rate of 10 rpm lower than the self-selected cadence, lasting for 10 s or more.

Pulmonary gas exchanges were measured breath-by-breath throughout the GXT by having the participants wear a face mask (7400 Vmask series, Hans Rudolph, Kansas City, MO, USA) attached to a portable gas analysis system (Cortex Metamax 3B; Cortex Biophysik, Leipzig, Germany). Before the test, the system was calibrated according to the manufacturer’s guidelines against known concentrations of cylinder gases (15% oxygen, 5% carbon dioxide) and a 3-L calibration syringe (5530 series, Hans Rudolph, Kansas City, MO, USA). Heart rates were monitored using a telemetry system with a wireless chest strap (Polar S810i; Polar Electro, Inc., Oy, Kempele, Finland) and were continuously measured through a link to the Cortex gas analysis system during the exercise test. The greatest $\dot{V}$O_2_ value (averaged every 10 s) measured during the GXT was recorded as the $\dot{V}$O_2_max value.

### 2.4. IPC Protocols

The LIPC and SHAM treatments were performed in supine position using 14-cm-wide blood pressure cuffs placed on the most proximal portions of the upper thighs. Bilateral occlusion was performed simultaneously on the right and left thighs. The cuff was rapidly inflated to 220 mmHg for the LIPC and 20 mmHg for the SHAM conditions, respectively, by means of a cuff inflator (CK-113P, Spirit Corp, Taipei, Taiwan). Absolute cuff pressure was used in SHAM to allow direct comparison with relevant studies [5,12,13,14]. This occlusion procedure was repeated 4 times, each separated by 5 min of reperfusion, as this protocol has been successfully applied in previous studies investigating the ergogenic effects of LIPC on exercise performance [5,6,13]. In RIPC treatment, the same blood pressure cuffs were placed bilaterally on the proximal parts of upper arms. Both cuffs were simultaneously inflated to 30 mmHg above systolic arterial pressure for 5 min, followed by 5 min deflation. The average cuff pressure in RIPC was 156 ± 7 mmHg. The cuffs were inflated and deflated 4 times in total, according with a previous study about the effect of RIPC on four sets of 30-s Wingate-based SIE performance [23]. In CON treatment, participants performed a 5-min standardized warm-up followed by a 5-min passive rest without any blood occlusion before SIE test.

### 2.5. Wingate-Based 6 × 30-s Sprint Interval Exercise

Prior to each trial, all participants performed a standardized warm-up comprising 5 min of submaximal cycling at 50 W (60–75 rpm), in which three bouts of maximal accelerations (approximately 5 s) at the end of the second, third and fourth minutes were performed. After the warm-up, participants rested for 5 min on the cycle ergometer. The SIE protocol involved participants to complete six sets (S1–S6) of 30-s Wingate-based SIE with a 4-min rest interval against the given load ([0.7 × body mass]/0.173) as fast as possible [25]. Thirty seconds before starting each sprint, participants were informed to ride at a moderate pedal cadence (50–60 rpm), from a seated position. Ten seconds before the initiation of each sprint, participants were instructed to increase pedaling rate to 80–85 rpm until given the signal to start pedaling maximally. During the last 2 s of the recovery period, participants were required to increase the pedaling rate to over 100 rpm (0 N) for the convenience of the subsequent 30-s cycling sprint test. At the end of the sprint, the participants remained seated on the bike for recovering between sets. Participants were given strong verbal encouragement by the same researchers throughout the entire trial.

The total work, peak (PPO) and mean (MPO) power outputs, and percentage decrement score (100 − [{total sprint power outputs/ideal sprint power outputs} × 100]) were calculated at each cycling sprint. The total sprint power was calculated as the sum of the peak/mean power outputs from all sprints. The ideal sprint power was defined as the number of sprints × highest peak/mean power output. The accumulated exercise time at ≥80%, 90%, and 100% $\dot{V}$O_2_max were also calculated during SIE.

### 2.6. Blood Sampling and Analysis

Capillary blood samples were taken by ear lobe puncture to evaluate lactate concentrations. The first blood samples were discarded, and the second blood samples (ca. 0.3 μL) were used to analyze the lactate concentrations using a lactate chemistry analyzer (Lactate Pro2, Arkray, Inc., Kyoto, Japan). To measure blood pH values, whole blood samples (ca. 1 mL) were drawn from an antecubital venous catheter and assessed by a blood gas analyzer (OPTI CCA-TS; OPTI Medical System, Inc., Roswell, GA, USA).

### 2.7. Statistical Analysis

The Shapiro–Wilk normality test was performed to determine the homogeneity of the sample. The performance data (total work, PPO, MPO, percentage decrement score, and accumulated exercise time), blood lactate and pH values were assessed using repeated-measures ANOVA. The LSD post-hoc test was applied if a significant difference was found. The effect size (Cohen’s *d*) was calculated by dividing the difference between the mean values of the conditions by the pooled SD. Cohen’s *d* of <0.5, 0.5–0.79, and ≥0.8 were considered as small, moderate, and large effects, respectively. The intraclass correlation coefficient (ICC) was used to assess the test–retest reliability of the SIE test in CON conditions. Statistical significance was set at *p* < 0.05 and all procedures were conducted using SPSS for Windows (Version 17, IBM SPSS Inc., Chicago, IL USA).

## 3. Results

**Figure 2:**
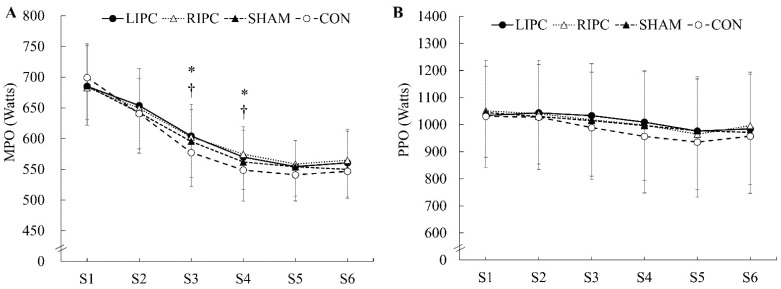
Effects of local (LIPC) and remote (RIPC) ischemic preconditioning on (**A**) mean power outputs (MPO) and (**B**) peak power outputs (PPO) during a sprint interval exercise (SIE) test. * *p* < 0.05, significant differences between LIPC and control (CON); † *p* < 0.05, significant differences between RIPC and CON; S1–S6, sprint 1–6.

The PPO (ICC = 0.93–0.98, *p* < 0.05) and MPO (ICC = 0.77–0.91, *p* < 0.05) at each sprint, and total work (ICC = 0.92, *p* < 0.05) demonstrated good to excellent test–retest reliabilities. The total work done during the SIE test differed significantly among treatments (*F* = 3.307, *p* < 0.05). The total work in the LIPC (+2.2%, *d* = 0.98) and RIPC (+2.5%, *d* = 1.10) conditions was significantly higher than that in the CON (Table 1). In Figure 2A, the MPO of the third and fourth sprint in LIPC (S3, +4.9%, *d* = 1.28; S4, +4.0%, *d* = 1.10) and RIPC (S3, +4.7%, *d* = 1.26; S4, +5.1%, *d* = 1.18) were significantly higher than those in the CON condition (*p* < 0.05). Table 1 also indicates that the percentage decrement scores of MPO in the LIPC (*d* = 1.08) and RIPC (*d* = 1.13) conditions were significantly lower than that in the CON condition (*F* = 3.534, *p* < 0.05). No significant interaction effect of PPO was found (*F* = 0.998, *p* > 0.05; Figure 2B); however, the percentage decrement score of PPO in the LIPC condition was significantly lower than that in the CON condition (*d* = 1.27, *p* < 0.05; Table 1).

There were no significant interaction effects in pH (*F* = 1.405, *p* > 0.05) and lactate (*F* = 1.035, *p* > 0.05) concentrations (Table 1). No significant differences in peak $\dot{V}$O_2_ were found during SIE among treatment conditions (LIPC vs. RIPC vs. SHAM vs. CON, 55.4 ± 7.7 vs. 55.6 ± 7.7 vs. 54.3 ± 10.2 vs. 55.7 ± 7.2 mL·kg^−1^·min^−1^, *F* = 0.296, *p* > 0.05). Moreover, there were no significant differences in the accumulated exercise time at ≥80% (*F* = 0.679, *p* > 0.05), 90% (*F* = 0.422, *p* > 0.05), and 100% (*F* = 0.741, *p* > 0.05) $\dot{V}$O_2_max during SIE test (Table 1).

## 4. Discussion

The purpose of this study was to examine the acute effects of IPC on the SIE performance in athletes. We hypothesized that both LIPC and RIPC could improve metabolic efficiency, and increase power output and fatigue resistance during SIE. To the best of our knowledge, no previous study has investigated the acute effects of LIPC and RIPC on the performance of six sets of SIE. The main finding of this study was that, for trained athletes, both LIPC and RIPC were effective in improving the total work of high-intensity interval sprints (ca. 2%) as well as the MPO during exercise (ca. 4%). In addition, compared to CON treatment, LIPC and RIPC treatment could also slow down the decline in power output. The reason for the escalation in SIE performance is likely that IPC increased metabolic efficiency during the high-intensity exercise.

Previous studies have reported that LIPC can improve the performance of six sets of 50-m swimming sprints with 3-min rest intervals [7], while RIPC can improve the PPO and MPO of four sets of Wingate-based sprints with 2-min rest intervals [23]. Our results were consistent with these findings, in that both LIPC and RIPC could improve the total work and the MPO of six sets of Wingate-based sprints with 4-min rest intervals. Previous studies have found that IPC can promote vasodilation as well as oxygen delivery and extraction by increasing the amount of vascular nitric oxide that can simulate vascular endothelial cells [5,26,27]. In addition, animal [28] and human trials [29] have discovered that IPC can facilitate the rate of PCr resynthesis. Reduced PCr availability has been considered as a limiting factor for power output recovery during multiple sprint exercises [30]. Although Paixao et al. [14] have reported that LIPC cannot improve the power output of three sets of Wingate-based sprint (with a rest interval of 10 min), it is more likely due to a long rest interval than to the effect of IPC on PCr resynthesis, as 6 min of passive rest after high-intensity exercise can restore PCr availability to 90% of the rest value [15]. Therefore, both LIPC and RIPC can improve the power output during SIE by promoting oxygen delivery and extraction as well as by facilitating PCr resynthesis during rest intervals.

Previous studies have suggested that LIPC cannot boost the percentage decrement score of 5–12 × 6-s cycling sprints [5,8,9]. In contrast, our study found that, compared to the CON condition, both LIPC and RIPC can increase the percentage decrement score. This inconsistent result may be attributed to different exercise patterns. First, among studies that have introduced multiple sets of repeated sprint exercises, Griffin et al. [6] reported that LIPC can escalate the percentage decrement score of three sets of exercises consisting of 6 × (15 + 15-m) shuttle sprints. In addition, a meta-analysis study by Salvador et al. [3] has indicated that, while IPC may have a >99% and a 57.9% chance of benefiting aerobic (>90 s) and anaerobic (10–90 s) exercise performance, respectively, its effect on the performance of sprints with a duration of less than 10 s is negligible. Similarly, other studies have pointed out that LIPC cannot improve the performance of 10–30 m sprints [10,11], while RIPC cannot enhance the power output and the fatigue index of a single 30-s Wingate sprint [19]. However, LIPC has been shown to improve the power output of both 60 s [13] and 3 min all-out [31] cycling sprint. Therefore, it appears that IPC can only facilitate repeated sprint exercises, with an extended single exercise time or total exercise time. Second, some studies have found that, for SIE, neither LIPC [14] nor RIPC [23] can consistently improve the fatigue index of every single 30-s Wingate sprint, in contradiction to our results. This is possibly due to different calculation methods, as these studies [14,23] only calculated the individual fatigue index of each sprint. As mentioned earlier, LIPC and RIPC can increase the total work as well as the percentage decrement score, thereby improving the fatigue resistance of SIE. Although this is probably associated with the promotion of muscle blood flow and PCr resynthesis during the rest interval, further research is required to explore the physiological and biochemical effects of LIPC and RIPC on the rest interval of SIE.

For further improvement in cardiopulmonary endurance, the exercise protocols must allow athletes to work for as much time as possible at an intensity close to or very close to $\dot{V}$O_2_max to expand the stimulating effect on the cardiopulmonary system [32]. The present study revealed that, compared to the CON condition, LIPC and RIPC did not change the accumulated time spent at high-intensity during SIE, and did not alter the blood lactate concentration or the H^+^ concentration post-SIE. This is consistent with the results of previous studies, which reported that the blood lactate concentration remains the same after high-intensity exercise if LIPC or RIPC is adopted [5,6,7,8,14,23,31]. Thus, LIPC and RIPC can improve the total work and the fatigue resistance of SIE without contributing to the acidic environment in the body, which indicates that LIPC and RIPC can improve the metabolic efficiency of SIE. Similarly, Incognito et al. [2] reported that, by increasing metabolic efficiency, IPC can improve the performance of subsequent exercises. Since LIPC and RIPC can escalate metabolic efficiency and total work, they can strengthen the training quality of a sprint interval training (SIT) program. This finding has partly supported the result of a study conducted by Paradis-Deschênes et al. [33], who found that the inclusion of LIPC in a 4-week SIT program could improve the aerobic and anaerobic performance of endurance athletes. However, at present, there has been no research on the chronic effect of RIPC interventions. Based on the results of this study, the inclusion of RIPC in the long-term SIT program in future studies is expected to provide additional adaptation benefits.

One limitation of this study was that participants were not completely blinded during the study. The presence of the placebo effect in IPC experiments remains controversial, with some studies finding [34,35] and others not findings evidence for this [7,36]. The use of placebo is a standard control component of most clinical trials, and it attempts to clarify the potential effect of treatment. It should be noted that a placebo effect relies on the assumption that participants believe (through conscious or unconscious cues) an intervention will change outcomes [37]. In general, previous IPC studies used low-pressure control conditions (e.g., 20 mmHg) as placebo conditions [5,12,13,14]. Although we did not inform participants which intervention we thought could improve their exercise performance, the participants could still distinguish the pressure difference between IPC and SHAM. The study of Marocolo et al. [35], who informed participants that both IPC and SHAM (20 mmHg cuff pressure) can increase exercise performance before starting the experiment, found that compared with CON, both IPC and SHAM could significantly increase the number of repetitions during the leg extension exercise. However, the study by Ferreira et al. [7], who told participants that both IPC and SHAM can enhance performance prior to experiment, using 1-min blood flow occlusions (200 mmHg cuff pressure) as SHAM to simulate the potential nocebo effect of IPC. The results indicated that SHAM induced similar levels of discomfort to IPC but comparable exercise performance outcomes to the CON [7]. In the study of Cheung et al. [36], they informed participants that the effects of IPC and SHAM on exercise performance were unclear before starting the study, and used the therapeutic ultrasound procedure as SHAM condition. They reported that 69% of participants believed that IPC would hinder exercise performance, whereas SHAM ultrasound could improve performance [36]. Nevertheless, the results indicated that only IPC could significantly increase time to exhaustion during incremental cycling test [36]. Therefore, the placebo/nocebo effects still seem to be a limitation of studies focusing on IPC and exercise performance.

Another limitation of this study was that the external validity and applicability of the results of this study are limited, as they can be influenced by the particular exercise mode as well as the training status, sex, and age of the participant. For example, participants in this study received 6 weeks of structured training using SIT 6 months before the experiment. Therefore, it needs to be verified whether the same promotion effect is observed when LIPC and RIPC are applied to untrained and obese individuals or diabetes patients, who are common research participants in SIT programs.

## 5. Conclusions

This study found that, for trained athletes, LIPC and RIPC were both ergogenic aids that could improve the performance and fatigue resistance of SIE. In addition, they did not alter the stimulus that SIE demonstrated on the cardiopulmonary system, which indicated that IPC could increase metabolic efficiency during high-intensity interval exercise. Further research is required to specify the possible mechanism by which IPC improves power output and delays fatigue during SIE.

## Figures and Tables

**Table 1:** Effects of local and remote ischemic preconditioning on exercise performance and physiological responses during sprint interval exercise test.

	LIPC (*n* = 15)	RIPC (*n* = 15)	SHAM (*n* = 15)	CON (*n* = 15)
Total work (kJ)	108.3 ± 8.9 *	108.4 ± 6.9 *	107.1 ± 8.6	106.0 ± 8.6
Percentage decrement score (%)				
PPO	5.4 ± 2.3 *	6.2 ± 3.1	6.5 ± 2.9	7.6 ± 3.8
MPO	11.9 ± 4.7 *	11.9 ± 4.6 *	13.1 ± 4.1	15.2 ± 5.3
Accumulated exercise time (s)				
$\geq 80\%\;\dot{V}$O_2_max	95.5 ± 52.7	99.9 ± 53.9	81.2 ± 62.0	95.2 ± 59.5
$\geq 90\%\;\dot{V}$O_2_max	34.5 ± 28.0	36.7 ± 33.9	28.9 ± 32.9	37.5 ± 40.1
$\geq 100\%\;\dot{V}$O_2_max	9.1 ± 11.7	10.5 ± 13.1	7.5 ± 11.5	11.6 ± 17.3
Lactate (mmol/L)				
Baseline	0.96 ± 0.21	1.01 ± 0.23	0.95 ± 0.27	0.95 ± 0.24
5 min after treatment	1.02 ± 0.24	1.04 ± 0.17	0.95 ± 0.15	-
5 min after SIE	11.09 ± 1.73	10.83 ± 2.02	10.62 ± 2.89	11.59 ± 2.21
pH				
Baseline	7.37 ± 0.02	7.39 ± 0.03	7.37 ± 0.02	7.38 ± 0.02
5 min after treatment	7.39 ± 0.02	7.40 ± 0.02	7.39 ± 0.02	-
5 min after SIE	7.20 ± 0.05	7.20 ± 0.05	7.20 ± 0.05	7.19 ± 0.05

LIPC, local ischemic preconditioning; RIPC, remote ischemic preconditioning; SHAM, sham; CON, control; PPO, peak power output; MPO, mean power output; $\dot{V}$O_2_max, maximal oxygen uptake; SIE, sprint interval exercise; * *p* < 0.05, compared with CON.

## Data Availability

Data sharing not applicable.
